# Recent Insight in Islet Amyloid Polypeptide Morphology, Structure, Membrane Interaction, and Toxicity in Type 2 Diabetes

**DOI:** 10.1155/2016/2535878

**Published:** 2015-12-07

**Authors:** Lucie Khemtemourian, Ehud Gazit, Andrew Miranker

**Affiliations:** ^1^Laboratoire des Biomolécules, Sorbonne Universités, UPMC Université Paris 06, 4 place Jussieu, 75005 Paris, France; ^2^Département de Chimie, Ecole Normale Supérieure-PSL Research University, 24 rue Lhomond, 75005 Paris, France; ^3^UMR 7203, Laboratoire des Biomolécules, CNRS, 75005 Paris, France; ^4^Department of Molecular Microbiology and Biotechnology, George S. Wise Faculty of Life Sciences, Tel Aviv University, 69978 Tel Aviv, Israel; ^5^Department of Molecular Biophysics and Biochemistry, Yale University, 260 Whitney Avenue, New Haven, CT 06520-8114, USA

The formation of protein amyloid deposits is associated with major human diseases including Alzheimer's disease, Parkinson's disease, Spongiform Encephalopathy, and type 2 diabetes mellitus (T2DM). Today, 382 million people live with diabetes. Diabetes is on the rise all over the world and countries are struggling to keep pace treating all these patients. Worldwide, one person dies of the consequences of diabetes (cardiovascular disease, kidney failure, and lower limb amputation) every 6 seconds, more than AIDS and malaria combined.

There are two types of diabetes. The first, type 1 diabetes mellitus, represents estimated 5–10% of the cases and results from the autoimmune destruction of the insulin-producing *β* cells in the pancreas, which leads to an absolute lack of insulin. The second, type 2 diabetes mellitus, represents estimated 90–95% of all diabetes cases and is characterized metabolically by hyperglycemia resulting from both insulin resistance and the relative lack of insulin secretion.

A hallmark of T2DM is the presence of extracellular amyloid deposits in the islet of Langerhans in the pancreas. These deposits are formed by the human islet amyloid polypeptide (hIAPP), a 37-residue peptide that is cosecreted and coproduced with insulin. Under normal conditions, the peptide hIAPP remains soluble but, in the pancreas of T2DM patients, the increase in peptide concentration and misfolding gives rise to oligomerization and to amyloid fibrils formation via a nucleation-dependent polymerization process. Studies suggest not only that the amyloid deposits are a minor epiphenomenon derived from the disease progression but that hIAPP aggregation induces processes that lead to the damage of the functionality and viability of *β* cells.

Despite considerable progress, there are still important outstanding issues in the field of islet amyloid. In this special issue, biophysical, physicochemical, and theoretical approaches are described (i) to define the structure and the orientation as well as the functions of hIAPP and (ii) to elucidate the molecular mechanism of aggregation of hIAPP. The influence of membranes, pH, and metal ions on hIAPP aggregation and hIAPP structure is also discussed. In addition, a recent methodology, sum frequency generation vibrational spectroscopy, is presented to investigate the aggregation of hIAPP on membrane surfaces. Finally, an overview of molecules that inhibit hIAPP fibril formation is given with emphasis on small molecules, natural molecules, and hIAPP variants.

